# Functional Analysis of a Polluted River Microbiome Reveals a Metabolic Potential for Bioremediation

**DOI:** 10.3390/microorganisms8040554

**Published:** 2020-04-12

**Authors:** Luz Breton-Deval, Ayixon Sanchez-Reyes, Alejandro Sanchez-Flores, Katy Juárez, Ilse Salinas-Peralta, Patricia Mussali-Galante

**Affiliations:** 1 Cátedras Conacyt - Instituto de Biotecnología, Universidad Nacional Autónoma de México, Avenida Universidad 2001, Colonia Chamilpa, Cuernavaca 62210, Morelos, Mexico; ayixon@ibt.unam.mx; 2 Unidad Universitaria de Secuenciación Masiva y Bioinformática, Instituto de Biotecnología, Universidad Nacional Autónoma de México, Cuernavaca 62210, Mexico; alexf@ibt.unam.mx; 3 Instituto de Biotecnología, Universidad Nacional Autónoma de Mexico, Cuernavaca 62210, Mexico; katy@ibt.unam.mx (K.J.); ilse@gmail.com (I.S.-P.); 4 Laboratorio de Investigaciones Ambientales, Centro de Investigación en Biotecnología, Universidad Autónoma del Estado de Morelos, Avenida Universidad 1001, Colonia Chamilpa, Cuernavaca 62209, Morelos, Mexico; mussali@uaem.com

**Keywords:** Apatlaco river, water pollution, superficial water, bioremediation, plastic biodegradation, biotechnology

## Abstract

The objective of this study is to understand the functional and metabolic potential of the microbial communities along the Apatlaco River and highlight activities related to bioremediation and its relationship with the Apatlaco’s pollutants, to enhance future design of more accurate bioremediation processes. Water samples were collected at four sampling sites along the Apatlaco River (S1–S4) and a whole metagenome shotgun sequencing was performed to survey and understand the microbial metabolic functions with potential for bioremediation. A HMMER search was used to detect sequence homologs related to polyethylene terephthalate (PET) and polystyrene biodegradation, along with bacterial metal tolerance in Apatlaco River metagenomes. Our results suggest that pollution is a selective pressure which enriches microorganisms at polluted sites, displaying metabolic capacities to tolerate and transform the contamination. According to KEGG annotation, all sites along the river have bacteria with genes related to xenobiotic biodegradation. In particular, functions such as environmental processing, xenobiotic biodegradation and glycan biosynthesis are over-represented in polluted samples, in comparison to those in the clean water site. This suggests a functional specialization in the communities that inhabit each perturbated point. Our results can contribute to the determination of the partition in a metabolic niche among different Apatlaco River prokaryotic communities, that help to contend with and understand the effect of anthropogenic contamination.

## 1. Introduction

Rivers are complex freshwater systems continuously connected to terrestrial environments, flowing towards an ocean, sea, lakes, or other rivers. These connections create dynamic riverine networks that experience a high flux of nutrients [1]. At present, rivers unusually transport high amounts of carbon and pollutant compounds as a result of human activities [2]. Given the variety of human activities, there is a complex mixture of chemical substances that flow into the waterway, such as heavy metals, hydrocarbons, higher amounts of organic matter, as well as other molecules such as chlorinated, nitroaromatic and organophosphate compounds [3]. The biogeochemistry of polluted rivers is unbalanced because the captured energy to support either biosynthesis or respiration is more than what the system requires. Additionally, the presence of toxic compounds modifies the microbial community present in water and sediments [4]. Currently, it is necessary to address water pollution from a practical and affordable approach. A way to achieve this is via bioremediation, which is the process used to reduce environmental contaminants by employing enzymes, microorganisms, plants, microbial metabolites, or other bioproducts [5,6]. However, some bioremediation strategies are not efficient due to the lack of understanding about specific microbes and their role in pollutant transformation. Since only 1% of microorganisms in a community are cultivable using traditional microbiology techniques, their characterization is very limited [7]. In contrast Next-Generation, Sequencing and Whole Metagenome Shotgun (WMS) approaches can be used to explore the total genomic content and discover innovative microbial metabolic functions in a sample. Additionally, a better taxonomic resolution can be achieved, in comparison to taxonomic profiles generated by the 16S rRNA gene sequencing [8,9]. Research related to microbial ecology of perturbed sites, including the complete characterization of microbial diversity, the metabolic functions of microbes and other factors that influence their metabolism, could be useful in determining the genetic pool of enzymes necessary for pollution tolerance and survival [10,11]. In some cases, polluted sites may already include microorganism species that tolerate or transform the contaminant. However, those species are not necessarily the most abundant, due to the lack of an appropriate carbon source [11]. The Apatlaco River is located in central México in the state of Morelos. It is 63 km in length and provides water to 10 state districts and an average of 2 million people [12]. The river receives 321 wastewater discharges, of which 49% come from industrial activities, 42% from the domestic sector, and just 9% from farming. As a result of these discharges, the river contamination is highly heterogeneous with chemical and microbiological components that may represent an epidemiological risk for the surrounding population [13,14]. Hence, it is necessary to find a feasible solution to treat the river water which supports an approximate population of 824,579 inhabitants and is essential for regional development. Concerned with this problem, this research aims to understand the functional and metabolic potential of the microbial communities in the Apatlaco River and highlight its relations with natural bioremediation capabilities. We hypothesize also that the recent history of pollution in the Apatlaco River has been shaping biodegradation functions, related to industrial pollutants.

## 2. Material and Methods

### 2.1. Study Site, Sample Collection, and Chemical Parameters Measured

**Figure 1:**
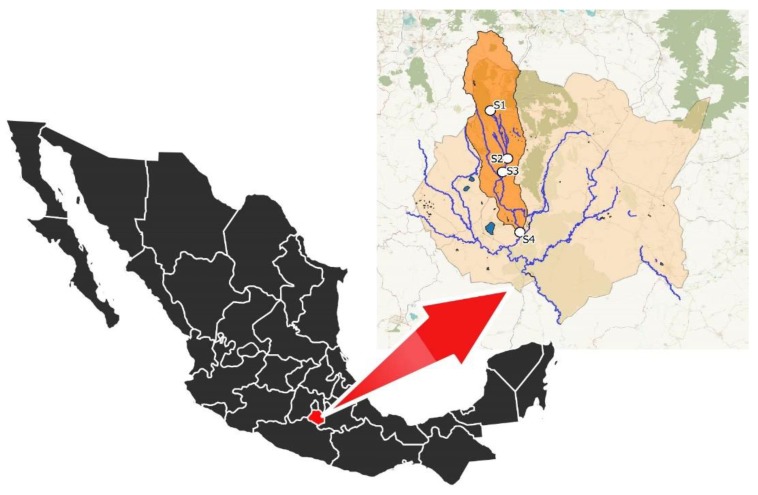
Map of México and the hydrology of Morelos. The Apatlaco basin is in orange.

Sampling was conducted every two months during dry season, 2018 (Oct–May) in order to span the entire cycle of hydrological variation. Water samples were collected at four sampling sites along the Apatlaco River (S1–S4), where S1 is our reference for clean water, (−99.26872, 18.97372) while S2 (−99.2187, 18.83), S3 (−99.23337, 18.78971) and S4 (−99.18278, 18.60914) were considered as human activity disturbed sites (Figure 1). At each sampling site, 10 different water samples of 1 L were collected, three of them were selected as independent samples for chemical analysis and each one was analyzed twice for a total of 6 technical replicates per site. Furthermore, all samples were pooled for metagenomic molecular analyses.

The samples were kept on ice and transported to the laboratory for further processing.

The HANNA multi-parametric instrument DR900 was used to measure the following parameters: dissolved oxygen (DO), total nitrogen (TN) and total phosphorus (TP). The chemical oxygen demand (COD) was determined with the colorimetric method using HACH digester DRB200 and the DR900 portable colorimeter, and total dissolved solids (TDS) were measured according to the protocol 2540 of the Standard Methods (Table 1) [15].

Four representative water samples were analyzed for metal content by atomic absorption spectrophotometry (908 AA, GBC), with background correction. All samples were put to a final volume of 100 mL. To ensure a satisfactory accuracy of the analysis, Standard Reference Material of National Institute of Technology and internal reference materials were used for precision, quality assurance and control for selected metal measurements. All the material used was previously washed with HNO_3_ ultra-pure (J.T. Baker) for 24 hrs. For each measurement, the average values of three replicates were recorded. Metal content is reported as mg/L (Zn, Cd, Pb, Cu, Mn, Cr).

Detection limits of the atomic absorption spectrophotometer are: 0.0005 mg/L for Zn, 0.01 mg/L for Pb, 0.0015 mg/L for Mn, 0.003 mg/L for Cr, 0.001 mg/L for Cu, 0.0004 mg/L for Cd and 0.005 mg/L for Fe.

### 2.2. DNA Extraction and Sequencing

DNA was extracted from water samples using a DNeasy PowerWater Kit (QIAGEN, Hilden, Germany). For each sample, an Illumina library was prepared from total DNA using the TruSeq kit v2 (Illumina, Inc., San Diego, CA, USA) following the manufacturer’s specifications with an average fragment size of 500 bp. The sequencing was performed on the NextSeq500 (Illumina, Inc., San Diego, CA, USA) platform with a 150-cycle configuration, generating paired-end reads with a length of 75 bp. Sequencing was deposited at the NCBI database under the Bioproject number PRJNA547779.

### 2.3. Bioinformatics Analysis

After performing a quality control analysis using the FASTQC program, low-quality sequences were removed using an in-house Perl script [16]. The taxonomic profiling was performed using the raw reads with the software MetaPhlan v2.0 [17], using the following parameters: --bt2_ps sensitive-local --min_alignment_len 95 --input_typefastq. For the metagenome assemblies, gene prediction and annotation, the raw reads were assembled using Megahit v1.1.3 [18], Metagenemark v3.36 [19] and Trinotate [20] v3.1.1, respectively. The parameters used for Megahit were: --k-min 25 --k-max 75 --k-step 10 -m 0.4 --no-mercy. The parameters used in Metagenemark were: -a -d -f G -m MetaGeneMark_v1.mod. For Trinotate, default parameters were used. GhostKOALA was used for KEGG’s annotation and K number assignment of metagenomic sequences [21].

### 2.4. Biodegradative and Metal Related Activities Prediction by Hidden Markov Models (HMM) Profiles

HMMER V 3.2.1 was used to search homologous sequences related to polyethylene terephthalate (PET) and polystyrene biodegradation functional domains, as well as for heavy metal related sequences (cadmium and lead). For this, we created custom profiles using a manually curated selection of representative sequences belonging to: (*i*) 11 well-characterized PET hydrolases [22], (*ii*) two sequences of styrene monooxygenase (StyA) functionally characterized, and (*iii*) for metals we selected well-known efflux transporters for Cd and Pb [23,24,25,26]. The construction of multiple alignments for each protein set was carried out by using Muscle v3.8 [27]. The generated data of selected markers is available at http://dx.doi.org/10.17632/2c8fhjb9kj.1. The alignments were manually curated and HMM profiles were generated using the hmm build command with default options (hmmbuild hmmfile_name alignfile.aln). The targets were the assembled contigs from each sample obtained from Apatlaco River S1, S2, S3, S4 sites containing a total of 499,126 contigs. We selected significant query matches using E-value threshold ≤ 0.001. We reported the associated taxonomy at genus level for each result, based on a Blastp using the same database to construct the HMM profiles.

### 2.5. Statistical Analysis

The microorganisms and chemical data sets were correlated by the exploratory multivariate statistical technique, principal component analysis (PCA) using R v3.5. The PCA derives linear combinations of the quantitative variables such as the relative abundance of the microorganism and the chemical analysis to reduce the dimensionality of the factors and explain the percentage of the variation amongst those variables [28,29].

An X^2^ test of independence was performed to examine the relation between water quality and the wastewater discharged sites. We set up a 2 × 4 contingency table with the frequencies in which each site showed values of water quality inside or outside the accepted normativity. Similarly, the relation between the abundance of KEGG molecular functions and the sampled sites was evaluated according to X^2^ test (11 × 4 contingency table). The KEGG overrepresented functions were assessed by a binomial test associated with S1 as a null hypothesis model. All cases were evaluated with a significance level of 0.05.

## 3. Results and Discussion

### 3.1. Pollution and Water Quality in the Apatlaco River Like a Selective Pressure to Autochthonous Microbial Communities

**Figure 2:**
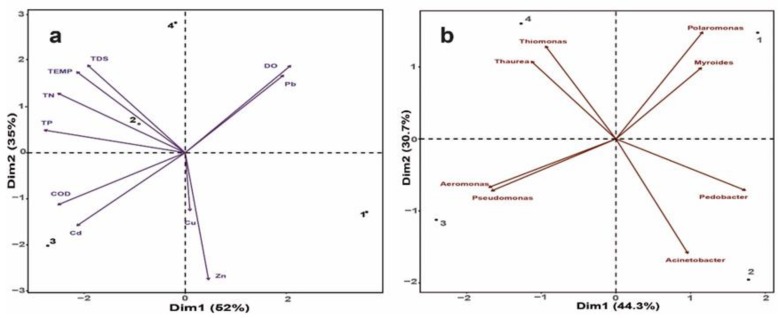
Principal component analysis (PCA). Where (**a**) explain the water quality variation is chemical factors and (**b**) explain the correlation with microorganism.

Daily, Apatlaco River receives 321 different discharges of wastewater from the manufacturing industry, domestic and agro-sector [13]. Our results showed that the water quality throughout the river is associated with the wastewater discharged sites (Table 1), (Figure 2) according to a chi-square test of independence (*X^2^* (3, *N* = 24) = 10.86, *p* = 0124). Water quality for sites S2 to S4 is generally low, while at the S1, the site at which the Apatlaco River is born, the quality is good according to the chemical analysis and the WHO water quality standards [30]. These results suggest linear contamination of the river as it comes into contact with the population and industrial settlements.

The PCA analysis (Figure 2a) showed that 52% and 38% of the water quality variation is explained in the first two principal components (PC), and the chemical factors which make a major contribution are Cd (19%), TP (19%), TN (15%) and COD (12%) in the first PC and TDS (20%), Pb (15%) and DO (12%) for the second PC. With regard to the locations, S1 is not related to any pollutant, while, the primary pollutants that affect S2 are total phosphorus (TP) and total nitrogen (TN). While S3 is near to a wastewater treatment plant and the site is impacted by the high levels of the chemical oxygen demand (COD) and Cd., S4, the last point, is affected by the highest levels of TDS as a result of an accumulation process and by a loss of the self-purification capacity of the river [31]. The changes in the water quality throughout the river also affect the microbial communities. Several studies have found that sewage discharges to the rivers enrich the microbial populations with pathogenic microorganisms, which usually do not live there and decrease the abundance of sulphate reducers, denitrifies and ammonium oxidizers. These microorganisms are vital to biogeochemical cycles and the self-purification capacity of the river [32]. Breton-Deval et al. [13] explored the microbial communities throughout the Apatlaco River (S1, S2, S3, S4) and found that polluted sites (S2, S3, S4) are enriched in *Acinetobacter, Arcobacter, Prevotella* and *Aeromonas*, all potential opportunistic pathogens; while the cleanest site (S1) is rich in planktonic bacterium widespread in freshwater ecosystems such as *Limnohabitans* and *Polaromonas.*

The summary of the metagenomic and bioinformatic analysis (Table 2) showed less genera assigned to the S1 than the other sites; perhaps the discharges enriched the sites S2-S4. The following results only concern the microorganisms present in S1-S4 with the potential to carry out bioremediation strategies accordingly to the KEEG analysis. Previous research related to the taxonomy profile of the whole community present at every site can be found at Breton-Deval et al., [13].

Our results suggest that pollution is a selective pressure which enriches the polluted sites with microorganisms with the metabolic capacities to tolerate and transform the contamination (Figure 2b). All sites along the river (S1–S4) have microorganisms that can be selected to carry out bioremediation activities according to the KEGG analysis and by experimental research, as reported elsewhere. However, every site has a unique profile of potential metabolic capacities related to the relative abundance of every microorganism (Table 3). These microorganisms are *Thiomonas*, which are able to accumulate, absorb and reduce heavy metals [33,34].

*Pseudomonas* is a ubiquitous microorganism able to degrade several pollutants such as hydrocarbons, phenol, pesticides and some metals such as chromium and cadmium [35,36]. *Myroides*, can tolerate high levels of Pb and Cu [37]. *Polaromonas* is able to degrade naphthalene, benzene, chlorobenzene and atrazine [38,39], *Acinetobacter* is able to degrade different compounds such as detergents, dyes, pesticides, hydrocarbons, clothianidin and cyprodinil [40,41,42], and *Aeromonas*, *Pedobacter* and *Thaurea* tolerate many metals including Zn, Cd, Co, Cu, Ni, Pb, Cr, Hg and Se [43]. However, the relative abundance of every genus is related to the chemical conditions of the site as a result of the metabolic capability of the microorganism, as we can see in Figure 2b. Furthermore, the genus which explain a high proportion of the variation inside the system are *Acinetobacter*, *Myroides* and *Thiomonas*.

### 3.2. The Functional Potential of the Microbial Community along the Apatlaco River

**Figure 3:**
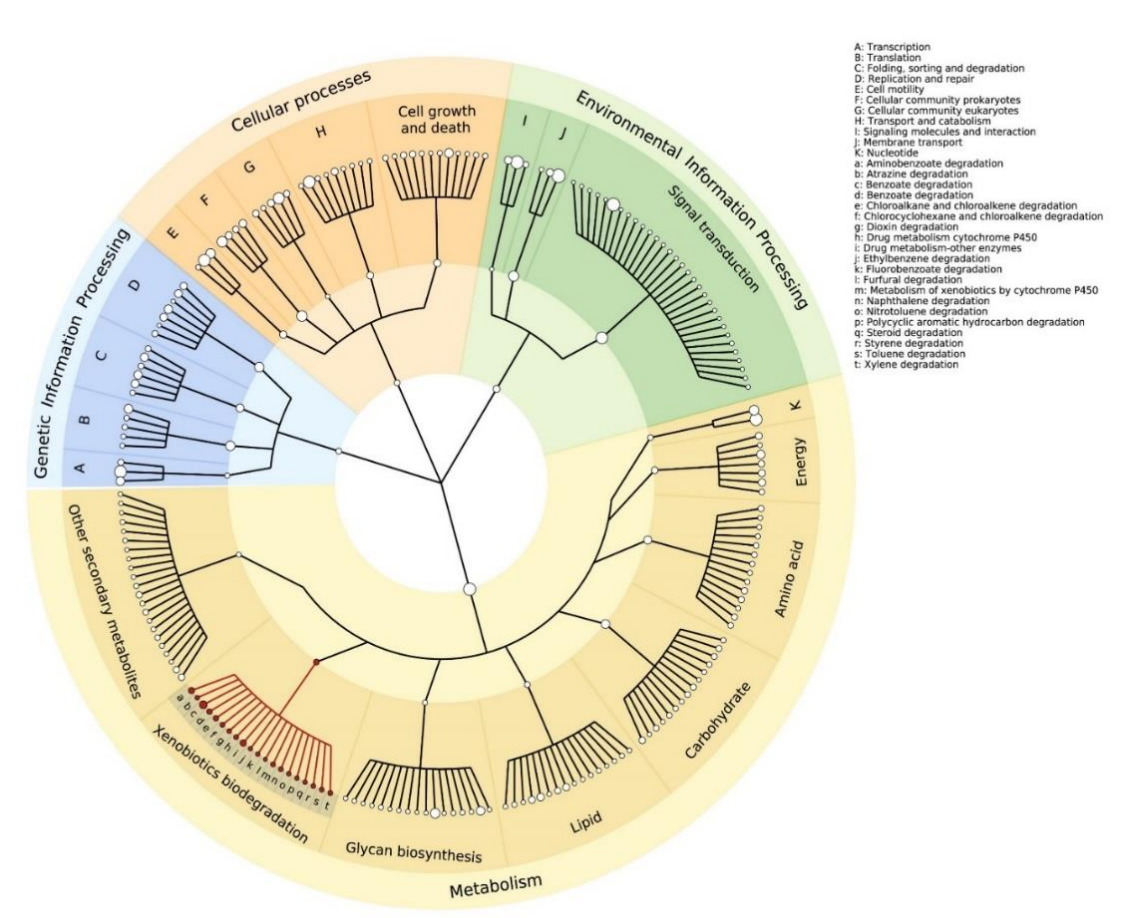
Abundance based cladogram of the KEGG molecular functions assigned to the predicted protein expression for all sampled sites. Xenobiotics biodegradation pathways found in the sites are in red and legend-specified in lowercase letters. The size of the nodes correlates with the abundance of annotated sequences for each pathway.

The functional gene profile revealed different patterns of molecular and cellular functions present in the river. The most abundant functional groups were related to housekeeping functions, such as the cellular and genetic process of energy, lipid or nucleotide metabolism (Figure 3). The presence of these patterns showed a regular distribution of the microbial activities of the community in every site. However, there is a statistical dependence among the predicted abundance of molecular functions and the sampled sites, according to Chi square test (*p* = 0.0, X^2^ = 277.05). According to KEGG annotation, all the sites (S1–S4) have genes related to xenobiotic biodegradation of the most common pollutants found in rivers, such as benzoate, toluene, styrene, dioxins, steroid, atrazine and chloroalkanes (Figure 3, highlighted in red). In addition, functions such as environmental processing, xenobiotic biodegradation and glycan biosynthesis are over-represented in S2, S3 and S4 compared with S1 (*p* = 0.000061, 0.0071, 0.0011, respectively), which suggests a functional specialization in the communities that inhabit each point. This can determine the partition in a metabolic niche among different prokaryotic communities that help to contend with anthropogenic contamination. This ultimately is possibly favorable in environments with dynamic resource availability. Furthermore, there are genes related to cellular functions such as the glycan biosynthesis and the biosynthesis of secondary metabolites that reflect the microbial community adaptations to stress situations such as a polluted environment [59].

Another way to explore the functional potential of the microbial community along the river is to find the enzymes involved in the pathways of the pollutants. Among the enzymes involved in the biodegradation of aromatic compounds at S1 were found dioxygenases and dehydrogenases, which can degrade Catechol, Biphenyl, Naphthalene and Phthalate; while S2 and S3 are both rich in dioxygenase and decarboxylating dehydrogenases involved in the degradation of Toluene, Fluorobenzoate, Xylene, Phenylpropanoate and Phenol. S3 also has monooxygenases that degrade Benzene. All of the earlier mentioned enzymes were also found at S4. These compounds are used to make lubricants, drugs, dyes, pesticides and rubbers. Given that an industrial park is located close to the river, the aforementioned presence of enzymes suggests that the industrial park does not have the necessary water plant to correctly treat its effluents and perhaps this constant discharge has selected the microbial community.

### 3.3. Microbial Genes and Enzymes Involved in the Degradation of Industrial Pollutants: Plastics and Heavy Metals and Metalloids

The Apatlaco River is a basin mostly polluted with solid plastic residues such as PET derivatives and polystyrene foams among others. Every year the basin receives on average 5 tons of plastics [60] that remain without significant biological alteration. Although some databases include information about xenobiotic degradation pathways and the genes and enzymes involved in their degradation, some pollutants, such as PET and polystyrene, are missing. In order to find enzymes involved in PET and polystyrene degradation, Hidden Markov Model strategies were applied to search for potential homologous biodegradative sequences in several metagenomes obtained from the river. As expected, we found PET hydrolase candidate’s gene coding sequences belonging to the superfamily of alpha/beta-hydrolases (pfamPF00561) (Table 4). Almost 50% of the sequences belong to the *Flavobacteriia* class. Interestingly, two sequences contain proteolytic domains suggesting similarities with the catalytic mechanisms of several peptidases.

Eleven sequences appear to be classic esterase-lipase proteins, related to ester hydrolysis in a broad xenobiotic substrate family. Danso et al. [22] retrieved two metagenomic PET hydrolase sequences from hmm probabilistic models that were functionally active in polycaprolactone and PET hydrolysis, supporting the approach of undermining the metabolic potential in environmental microbiomes to degrade priority pollutants. Several studies suggest that the degradation of polystyrene by bacteria occurs due to individual oxidizing units of styrene, through monooxygenase activities [61]. Six oxygenase (StyA) candidate’s genes were found (Table 4). Homologous sequences in *Pseudomonas* and *Rhodococcusopacus* [62,63] are responsible for activation in aerobic bacterial styrene degradation. We hypothesized that these metagenomic sequences could support the Apatlaco River’s microbial communities in the biodegrading of PET and polystyrene.

Other compounds that are missing in most databases include heavy metals, and this is because some metals such as Cu, Zn and Fe are protein cofactors while other metals such as Cd, Ag and Pb do not play a role in bacterial metabolism [64]. However, even at deficient concentrations, non-essential metals or essential metals at high levels are toxic for microorganisms, and it can be difficult to distinguish between the pathways involved in metabolism and the different strategies that microorganisms employ to deal with heavy metals and metalloids. Microorganisms can adsorb metals, change their speciation to less harmful compounds, and mineralize [65]. Biosorption is mostly carried out by proteins called metallothioneins while speciation is a common reduction mechanism; some examples are Cr^6+^ to Cr^3+^, AsO_4_^3−^ to AsO_3_^3−^, Hg^2+^ to Hg^0^ [66].

The Aplataco’s water contains some metals; however, only Pb and Cd levels were above the national standards (Table 1). Cd and Pb are metals that do not have any biological function and are very toxic because they may affect the renal, hematologic and nervous systems. However, some reported bacteria can tolerate 1350 mg/L of Cd and 1900 mg/L of Pb [67]. The genes involved in Cd resistance are RND (resistance nodulation cell division protein family) exporters composed of the following proteins: CzcA, CzcB, CzcZ, CzcN, CzcD, CzcR and CzcS, which form transport systems that are able to export ions such as Zn^2+^, Co^2+^ and Cd^2+^ across the membranes. The application of the Hidden Markov Model to find homologous sequences of these proteins allowed the association with some microorganisms present in the river, with the ability to tolerate Cd and Pb. The number of species identified at each sample site is around five, except for S3 where 11 Cd tolerant species were found with a particular abundance of *Flavobacterium* (Table 5). This same point has the highest level of Cd of the whole river (0.19 mg/L). It is possible that the elevated level of Cd stimulates a more significant presence of the microorganism. In the case of Pb, the genes involved in Pb resistance are metal-transporting ATPase for Cd, the ATPase for Cu+ CopA, the ATPase ZntA, and the metallothionein protein SmtA [66] (Table 4). SmtA has been reported in *Synechococcus*, and *Pseudomonas* [66], while CopA and ZntA have been described in *E. coli* and *Enterococcus hirae* [68]. Our results identified microorganisms that have not been previously reported, such as *Limnohabitans*, *Cellvibrio*, *Polynucleobacter* or *Azoarcus*, which can tolerate Pb.

## 4. Conclusions

Our result allowed us to identify the microorganisms present along the Apatlaco River, with metabolic potential to carry out bioremediation activities, of the following genera: *Thiomonas*, *Polaromonas*, *Pedobacter*, *Myroides*, *Pseudomonas*, *Acinetobacter*, *Aeromonas* and *Thauera*. Furthermore, enzymes involved in the degradation of several priority pollutants were predicted. The differential distribution of biodegradative functions along the river implies different ecological niche development related to the degradation of xenobiotics, which would be necessary for studies of nutritional selection or isolating potential candidates for bioremediation. Although an oligotrophic stage prevails in the studied microbial communities of the Apatlaco River, the points analyzed seem to show some specialization concerning energy metabolism, as well as the potential to obtain new biocatalysts for biodegradation of emerging pollutants such as plastic wastes or reducing agents of concern to human health. Currently, we are carrying out gene expression studies on this site to understand what metabolic functions are active in the community. It is worthy to note the identification of microorganisms that have not been previously reported as Pb tolerant (*Limnohabitans*, *Cellvibrio*, *Polynucleobacter*, or *Azoarcus*), which deserve further study.

## Figures and Tables

**Table 1:** Measurements of physicochemical parameters along the Apatlaco River.

Pollutants	S1	S2	S3	S4
COD (mg/L)	80 ± 9.36	270 ± 23. 35	480 ± 31	110 ± 16
TN (mg/L)	6 ± 1.48	31 ± 6.74	38 ± 5.78	45 ± 6.45
DO (mg/L)	7 ± 0.59	4 ± 0.38	2 ± 1.19	7 ± 1.98
TP (mg/L)	0.77 ± 0.22	9 ± 2.65	16 ± 2.45	9 ± 1.47
TDS (mg/L)	62 ± 1.77	206 ± 9.75	299 ± 10.35	756 ± 20.67
Temp (°C)	17 ± 1.56	22 ± 1.10	23 ± 1.21	26 ± 2.25
Zn (mg/L)	0.20 ± 0.02	0.16 ± 0.02	0.20 ± 0.02	0.13 ± 0.01
Cd (mg/L)	0.07 ± 0.02	0.16 ± 0.01	0.19 ± 0.01	0.16 ± 0.01
Pb (mg/L)	3.95 ± 0.22	2.53 ± 0.27	2.24 ± 0.08	4.35 ± 0.15
Cu (mg/L)	0.99 ± 0.04	0.46 ± 0.02	1.15 ± 0.06	0.88 ± 0.03
Mn (mg/L)	<0.0015	<0.0015	<0.0015	<0.0015
Cr (mg/L)	<0.003	<0.003	<0.003	<0.003

**Table 2:** Summary of the metagenomic and bioinformatic analysis.

Parameter	S1	S2	S3	S4
**Total filtered pair-end reads**	41771406	64325748	64321034	47153792
**Assembly size (Mb)**	42.716	82.952	78.809	48.360
**Total contigs**	45488	103533	114279	59123
**N50/L50**	1959/4015	1626/9243	1165/14	1694/5245
**Total predicted genes**	75,951	164,571	165,377	93,227
**Total annotated genes**	26,910	35,597	44,547	28,939
**Total tax. Assig. (genus)**	73	108	140	136

**Table 3:** Microorganisms with bioremediation potential.

Microorganism	Rel. Abundance (%)				Compounds	Pathogen Ref.	
	S1	S2	S3	S4			
*Thiomonas* sp.	1.17	0.75	1.30	4.63	As, S,	No	[6,7,8,34]
*Polaromonas* sp.	1.74	0.41	0.24	0.73	Hg, As, alkanes, Pyrene	No	[23,44,45,46]
*Pedobacter* sp.	1.33	2.46	0.24	0.80	As(V)	opportunistic	[47]
*Myroides* sp.	27.2	5.19	6.20	4.03	CN^−^, SCN^−^ Organic matter	opportunistic	[24,48]
*Pseudomonas* sp.	0.72	1.77	5.07	7.58	Pb +2, Ni +2, Cu +2, Cr +3, NO_3_, detergents, dyes, pesticides, hydrocarbons	opportunistic	[25,36,49,50]
*Acinetobacter* sp.	0.30	50.2	5.79	0.72	Hydroxydioxane, Cr, Clothianidin, cyprodinil,	yes	[40,51,52,53]
*Aeromonas* sp.	0.29	0.25	2.03	0.54	As, Cu, Fe, Ni, Zn, Mn (II), dyes, PHAs,	yes	[54,55,56,57,58]
*Tavera* sp.	0.16	0.15	0.36	1.29	Zn, Cd, Co, Cu, Ni, Pb, Cr, Hg, Se	No	[43]

**Table 4:** Putative genes and enzymes involved in the degradation of PET and Polystyrene in the Apatlaco River’s metagenome.

DEGRADATION PATHWAYS GENE_ID	Site	Target Compound	Enzymes	Taxonomic Assignation
MT023458	S1	PET	S9 family peptidase	*Flavobacteriia bacterium*
MT023459	S1			
MT023460	S3		OsmC family protein Esterase	*Proteobacteria*
MT023461	S1		Alpha/beta hydrolase	*Bordetella flabilis*
MT023462	S2		dipeptidyl aminopeptidase	*Sphingobacteriales bacterium*
MT023463	S4		alpha/beta hydrolase	*Bacteroidia bacterium*
MT023464	S2			
MT023465	S4			
MT023466	S3			
MT023467	S3		alpha/beta hydrolase	*Lutibacter* sp.
MT023468	S1		alpha/beta hydrolase	*Flavobacterium aquicola*
MT023469	S2		alpha/beta hydrolase	*Flavobacterium aquicola*
MT023470	S3		alpha/beta hydrolase	*Flavobacterium aquicola*
MT023471	S1	Polystyrene	kynurenine 3-monooxygenase	*Flavobacteria bacterium*
MT023472	S1		FAD-dependent oxidoreductase	*Chitinophagaceae bacterium*
MT023473	S2		Ferredoxin	*Aurantimicrobium* sp.
MT023474	S2		FAD-dependent monooxygenase	*Flavobacteriia bacterium*
MT023475	S2		Ubiquinone biosynthesis protein UbiH	*Coxiellaceae bacterium*
MT023476	S4		Ferredoxin	*Aurantimicrobium* sp.

**Table 5:** Annotation of genes and microorganisms involved in tolerance to Cd and Pd predicted by HMM profiles using the cobalt-zinc-cadmium resistance protein CzcA and the 2-isopropylmalate synthase for Cd, and the P-type Cu2+ transporter, P-type Cu+ transporter and Zn2+/Cd2+-exporting ATPase for Cd.

Degradation Pathway Gene_ID	Site	Target Compound	Taxonomic Assignation
MT023477	S1	*Cd*	*Flavobacterium branchiophilum*
MT023478	S1		*Methylotenera versatilis*
MT023479	S1		*Methylotenera versatilis*
MT023480	S1		*Emticiciaoligo trophica*
MT023481	S1		*Azospirillum* sp. *B510*
MT023482	S1		*Dechloromonas aromatica*
MT023483	S1		*Sphingopyxis* sp. *QXT-31*
MT023484	S1		*Flavobacterium branchiophilum*
MT023485	S2		*Methylotenera mobilis*
MT023486	S2		*Chryseobacterium taklimakanense*
MT023487	S2		*Polynucleobacter duraquae*
MT023488	S2		*Cytophaga hutchinsonii*
MT023489	S2		*Acinetobacter schindleri*
MT023490	S2		*Myroides odoratimimus*
MT023491	S2		*Acinetobacter schindleri*
MT023492	S3		*Acidovorax citrulli*
MT023493	S3		*Flavobacteriuman huiense*
MT023494	S3		*Flavobacterium commune*
MT023495	S3		*Flavobacterium columnare*
MT023496	S3		*Chryseobacterium taklimakanense*
MT023497	S3		*Azospira oryzae*
MT023498	S3		*Polynucleobacter duraquae*
MT023499	S3		*Myroides* sp. *A21*
MT023500	S3		*Azospira oryzae*
MT023501	S3		*Flavobacterium commune*
MT023502	S3		*Sulfurimonas autotrophica*
MT023503	S4		*Methylotenera mobilis*
MT023504	S4		*Runella slithyformis*
MT023505	S4		*Polynucleobacter duraquae*
MT023506	S4		*Acidovorax citrulli*
MT023507	S4		*Shewanella* sp. *ANA-3*
MT023437	S1	*Pb*	*Flavobacterium johnsoniae UW101*
MT023438	S1		*Limnohabitans* sp. *63ED37-2*
MT023439	S1, S2		*Limnohabitans* sp. *103DPR2*
MT023440	S1, S2		*Limnohabitans* sp. *103DPR2*
MT023441	S2		*Azoarcus olearius*
MT023442	S2		*Polynucleobacter asymbioticus*
MT023443	S2		*Acinetobacter johnsonii*
MT023444	S2		*Acinetobacter schindleri*
MT023445	S2		*Azoarcus olearius*
MT023446	S3		*Acidovorax* sp. *1608163*
MT023447	S3		*Alicycliphilus denitrificans BC*
MT023448	S3		*Alicycliphilus denitrificans BC*
MT023449	S3		*Chryseobacterium taklimakanense*
MT023450	S3		*Acinetobacter johnsonii*
MT023451	S4		*Cellvibrio* sp. *PSBB006*
MT023452	S4		*Polynucleobacter asymbioticus*
MT023453	S4		*Limnohabitan* ssp. *63ED37-2*
MT023454	S4		*Beta proteobacterium CB*
MT023455	S4		*Limnohabitan* ssp. *63ED37-2*
MT023456	S4		*Azoarcus olearius*
MT023457	S4		*Acinetobacter johnsonii*
